# Virulence Variability of *Puccinia triticina* in the Western Region of the High-Latitude Asian Spring Wheat Belt

**DOI:** 10.3390/jof12100774

**Published:** 2026-10-15

**Authors:** Elena Gultyaeva, Evsey Kosman, Ekaterina Shaydayuk, Ekaterina Shreyder, Vladimir Shamanin, Alexey Morgounov, Igor Kushnirenko, Anastasiya Kazak, Elena Filippova, Anastasiya Chernook

**Affiliations:** 1 All Russian Institute of Plant Protection, Shosse Podbelskogo 3, St. Petersburg 196608, Russia; eshaydayuk@bk.ru; 2 Institute for Cereal Crops Improvement, School of Plant Sciences and Food Security, George S. Wise Faculty of Life Sciences, Tel Aviv University, Tel Aviv 69978, Israel; kosman@tauex.tau.ac.il; 3 Chelyabinsk Scientific Research Institute of Agriculture, Timiryazevskiy 456404, Russia; 4 Agronomy Department, Omsk State Agrarian University named after P.A. Stolypina, Omsk 644008, Russia; vp.shamanin@omgau.org; 5 All-Russia Research Institute of Agricultural Biotechnology, Moscow 127550, Russia; alexey.morgounov@gmail.com (A.M.); irbis-sibri@yandex.ru (A.C.); 6 Agricultural Institute, University of Tyumen, Tyumen 625003, Russia; 7 Ural Federal Agrarian Scientific Research Centre, Ural Branch of the Russian Academy of Sciences, Ekaterinburg 620142, Russia

**Keywords:** leaf rust, *Lr* resistance genes, northern Kazakhstan, population structure, Russia, Ural, Western Siberia

## Abstract

A comprehensive virulence survey of *Puccinia triticina* collections was conducted in 2025 across four regions of Asian Russia and northern Kazakhstan to analyze the extent of differentiation among the regional pathogen populations. Leaves with uredinia were collected on a standardized set of spring wheat genotypes from the Kazakhstan–Siberia Network (KASIB-26). A total of 195 single-pustule isolates (34 from Chelyabinsk, 24 from Kurgan, 52 from Tyumen, 43 from Omsk, and 42 from northern Kazakhstan) were tested for the infection type on the standard set of twenty Thatcher near-isogenic wheat lines. None of the 20 *Lr*-genes represented in the differential lines proved to be highly effective. New virulences to *Lr24* and to the gene combination *Lr9* + *Lr26* were first revealed in the studied area. In total, 22 races were identified. Two races were identified across all five regions, one in three regions, and four in two regions. Alongside traditional races in the northwesternmost wheat-growing territories in Asia, new races with previously absent virulences were detected in 2025. Distinct pathogen groups were identified within the Omsk and Tyumen regions, and a highly unique race was found in Kazakhstan. Nevertheless, the overall population structure remained stable during the last decade, with a predominant combination of highly virulent races persisting across the whole territory.

## 1. Introduction

Spring bread wheat (*Triticum aestivum* L.) is a major cereal crop cultivated extensively in Russia and Kazakhstan. In Russia, production is concentrated in the Volga region, the Urals, and Western Siberia [1], while in Kazakhstan it is predominantly grown in the northern regions [2]. The productivity of spring wheat is constrained by a range of biotic factors, including fungal pathogens, insect pests, and weeds. Among these, leaf rust, caused by the obligate biotrophic fungus *Puccinia triticina* Erikss., represents one of the most widespread and economically significant foliar diseases in spring wheat-growing areas, and it occurs regularly in both countries.

In 2020, CIMMYT established a joint breeding program with northern Kazakhstan and Western Siberia, formalized as the Kazakhstan–Siberia Network for Spring Wheat Improvement (KASIB). Participating institutes span Russia (from the Volga to Eastern Siberia) and Kazakhstan (north to south) [2]. The network conducts multi-location trials for yield, quality, and disease resistance using exchanged materials. Shuttle breeding with CIMMYT was implemented to incorporate resistance to multiple diseases, including leaf rust, while preserving local adaptation, drought tolerance, and grain quality. Each participating organization contributes two to four promising genotypes, which are evaluated over a two-year period across all trial locations. This program represents a model framework for germplasm evaluation and disease surveillance. Importantly, numerous elite lines developed through this network have been included in the State Registers of Breeding Achievements and are now commercially grown in both Russia and Kazakhstan. With leaf rust an ongoing problem for the production of spring wheat in Russia and Kazakhstan up to the mid-2010s, research and breeding institutes gave priority to developing new resistant cultivars, as there was initially a low proportion of resistant material identified. With time, some progress has been achieved, and the dynamic of increasing leaf rust-resistant genotypes in the KASIB network remained stable in 2020 [3].

The screening strategy for leaf rust encompasses three complementary components: field-based adult-plant evaluations, greenhouse seedling assays using pathotypes of known virulence, and molecular marker analysis for the detection of rust resistance genes. The integration of these confirmatory approaches has substantially enhanced the reliability and efficiency of resistance characterization across both the seedling and adult-plant developmental stages. Sustained progress in wheat breeding for rust resistance, however, necessitates continuous monitoring of resistance gene effectiveness and the impact of newly deployed cultivars on pathogen virulence. In this regard, parallel studies on the virulence of leaf rust populations and other diseases are being undertaken [4,5].

Surveys conducted in the early 2020s documented a suite of resistance genes effective against leaf rust (*Lr24*, *Lr28*, *Lr29*, *Lr39*, *Lr45*, *Lr47*, *Lr51*, *Lr6Agi1*, *Lr6Agi2*, and *LrAsp*) in spring wheat-producing regions of Russia and Kazakhstan [2,3]. Notably, the resistance conferred by *Lr9* and *Lr19* has been eroded in areas where these genes are extensively deployed, including the Volga region, the Urals, Siberia, and northern Kazakhstan. Nonetheless, evidence suggests that pyramiding these ineffective genes with *Lr26* and other sources can restore functional resistance.

A comprehensive assessment of *P. triticina* population diversity in the West Asian part of Russia and northern Kazakhstan was undertaken in 2016 and 2017, based on both virulence phenotyping and microsatellite genotyping [4,5]. Leaf rust samples were obtained from wheat accessions evaluated within the KASIB-16 network at multiple locations across Russia (Omsk, Chelyabinsk, Kurgan) and northern Kazakhstan (Akmolinsk, Karaganda, Pavlodar). The high concordance between virulence profiles and molecular markers strongly supports the hypothesis of a single, genetically cohesive *P. triticina* population spanning this region. These findings are corroborated by extended virulence monitoring of Russian *P. triticina* populations over the period 2000–2024, which has consistently revealed a high degree of similarity among isolates originating from the West Asian part of Russia [6,7].

In most studies of leaf rust pathogen populations, isolates are sampled from diverse cultivars grown across geographically distinct regions. The deployment of genetically heterogeneous host varieties at different locations can confound virulence analyses and complicate the inference of population structure. By standardizing sample collection on a common set of wheat cultivars, the influence of host genotype on race composition can be minimized. The Kazakhstan–Siberia Network, which evaluates a uniform panel of spring wheat varieties across multiple sites in Russia and Kazakhstan, therefore provides a unique opportunity for a more refined and unbiased characterization of *P. triticina* population structure in this region. The present study reports the results of a comprehensive survey of *P. triticina* populations conducted in 2025 across four regions of Asian Russia and one region of northern Kazakhstan. The primary objectives were (i) to characterize the virulence and pathotype variability and structure of *P. triticina* collections from geographically distant locations, using a standardized set of spring wheat genotypes from the Kazakhstan–Siberia Network (KASIB-26), and (ii) to compare the regional pathogen populations and infer the extent of their genetic differentiation.

## 2. Materials and Methods

### 2.1. Regions and Sampling

A new set of spring wheat accessions tested under the KASIB program in 2025–2026 included 48 spring wheat accessions, of which 18 originated from six breeding institutions in Kazakhstan and 21 from nine Russian institutions. The varieties Pamyati Azieva, Astana 2, Omskaya 35, and Saratovskaya 29 were included as international standards. Leaf rust symptoms were identified on 35 of the studied wheat accessions across the surveyed locations. Disease severity on the wheat accessions used as sources of leaf rust inoculum in the Russian regions is shown in Table 1, where it can be seen that it was much lower in northern Kazakhstan. Leaf rust severity was scored visually using the Peterson scale [8]. The main criterion was the maximum flag leaf infection severity recorded during the peak of disease development.

**Figure 1:**
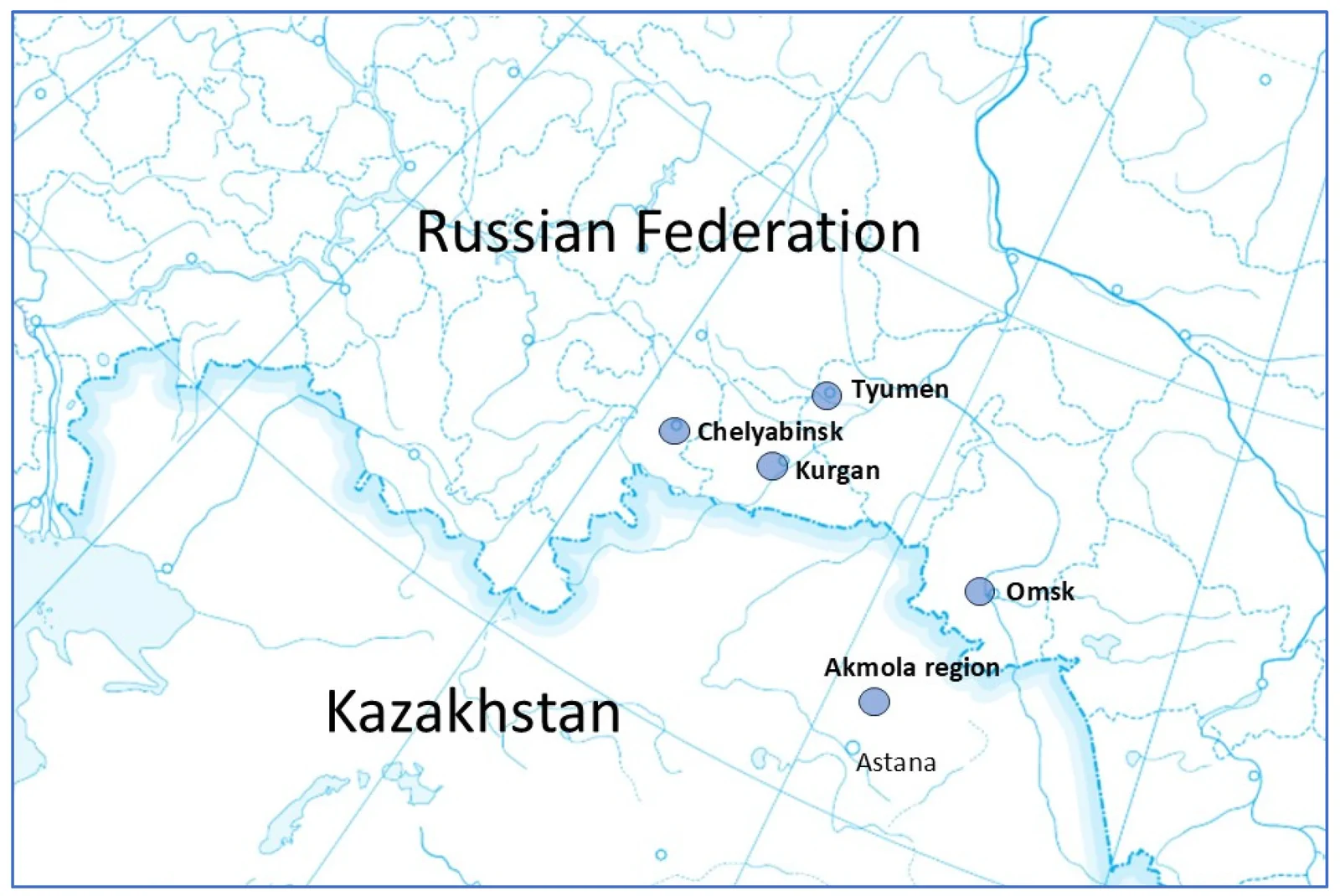
Collection sites of *P. triticina* in Russia and Kazakhstan.

Leaf rust samples were collected from five geographically distant locations: four in the Russian Federation (Kurgan, Chelyabinsk, Tyumen, and Omsk) and one in northern Kazakhstan (Akmola region) (Figure 1). In all five sites, *P. triticina* isolates were recovered from only two wheat accessions: Lutescens 22144 (No. 15) and 434/sp2/21 (No. 16). For the remaining accessions, the number of locations from which samples were obtained varied: 7 accessions were represented from four locations, 11 from three locations, and 10 from two locations (Table 1). The Russian sampling locations—Kurgan, Chelyabinsk, Tyumen, and Omsk—are situated in the Asian part of the Russian Federation, within the broader region of Northern Asia. Chelyabinsk region is positioned at the Europe–Asia divide and is classified as part of the Southern Urals. The Kurgan region occupies the transitional zone between the Urals and Western Siberia, commonly referred to as the Trans-Urals. The Tyumen and Omsk regions are both located in the southern part of Western Siberia. The Akmola region is part of Northern Kazakhstan and geographically belongs to Central Asia. All these regions can be considered high-latitude northwestern territory of wheat growing in Asia.

### 2.2. Virulence Analysis

Establishment of single-pustule isolates. 

Infected leaves were placed in paper bags, air-dried at room temperature for 1–2 days, and stored at 4 °C until processing. Reanimation of *P. triticina* urediniospores was carried out following the previously described protocol [7,9]. Urediniospores were scraped from infected leaves of each spring wheat accession and suspended in a small volume of water containing Tween 20. The susceptible wheat variety Michigan Amber was then inoculated by rubbing the leaves with the urediniospore suspension. To prevent cross-contamination, plants inoculated with different isolates were separated using perforated cellophane bags. Following inoculation, plants were placed in a dark dew chamber for 17–24 h and subsequently incubated at 20–22 °C with a 16 h light/8 h dark photoperiod. At 8–10 days post-inoculation, leaves were trimmed to ensure each leaf carried a single uredinium. Urediniospores from individual pustules were collected using a vacuum suction micro-collector, starting 14 days after inoculation and repeated every 3–4 days until leaf desiccation.

Host differential set and phenotyping.

Twenty Thatcher near-isogenic wheat lines with leaf rust resistance genes *Lr1* (RL6003), *Lr2a* (RL6000), *Lr2b* (RL6019), *Lr2c* (RL6047), *Lr3* (RL6002), *Lr3bg* (RL6042), *Lr3ka* (RL6007), *Lr9* (RL6010), *Lr11* (RL6053), *Lr14a* (RL6013), *Lr14b* (RL6006), *Lr15* (RL60052), *Lr16* (RL6005), *Lr17* (RL6008), *Lr18* (RL6009), *Lr19* (RL6040), *Lr20* (RL6092), *Lr24* (RL6064), *Lr26* (RL6078), and *Lr30* (RL6049) were inoculated 8–10 days after planting. The Thatcher variety was included as a susceptible control. For virulence testing, spores of each single-pustule isolate were suspended in a light mineral oil (NOVEC 7100) and sprayed onto the differential host set. Following inoculation, plants were allowed to air-dry for a minimum of 1 h to ensure complete oil evaporation, and subsequently incubated and maintained under the conditions described above. Virulence characterization was carried out on both bulk urediniospore inoculum multiplied from each wheat accession and two single-pustule isolates derived from the same samples. For subsequent data analyses, only the data obtained from the single-pustule isolates were considered, thereby excluding potential genotypic heterogeneity present in the bulk inoculum.

Infection types were scored 12 days post-inoculation according to the 0–4 scale described by Mains and Jackson [6,10]. Isolates inducing infection types 0–2 were classified as avirulent, whereas those producing types 3–4 were considered virulent. In cases where the infection response was unclear, the inoculation was repeated.

Virulence phenotypes were treated as binary variables, with avirulence and virulence coded as 0 and 1, respectively. The differential host panel was composed of five groups, each comprising four near-isogenic lines carrying individual *Lr* resistance genes. Set 1: *Lr1*, *Lr2a*, *Lr2c*, and *Lr3*; set 2: *Lr9*, *Lr16*, *Lr24*, and *Lr26*; set 3: *Lr3ka*, *Lr11*, *Lr17*, and *Lr30*; set 4: *Lr2b*, *Lr3bg*, *Lr14a*, and *Lr14b*; set 5: *Lr15*, *Lr18*, *Lr19*, and *Lr20*. Adapted North American nomenclature (hexadecimal code) was used for phenotype designations [11,12].

### 2.3. Data Analysis

Races of *P. triticina*, their relative abundances and distribution, and virulence frequencies to *Lr* genes were analyzed. Descriptive parameters, such as virulence frequency and relative virulence complexity (RVC) [13], were calculated for the regional collections and some other groups of isolates.

The structural variability of the *P. triticina* collections was analyzed based on the simple mismatch dissimilarities (*sm*) between virulence profiles of isolates and races. The assignment-based *KW* dispersion within and *KB* distance between collections were calculated [14,15]. The permutation test (1000 random partitions) for differentiation statistics ${dif}_{KW}$ (Equation (1) in [6] for the *KW* dispersion (see also Equation (13) in [15]) was applied to estimate differentiation among the *P. triticina* regional collections and groups.

The effective number of different isolates (*ENDI*) within a collection was estimated according to Equations (5) and (6) in [16] for M=KW; *ENDI* values range from 1, if all isolates are identical, to an actual number of isolates when they are absolutely different. The normalized version *nENDI* of this indicator was calculated (Equation (3) in [17]) to compare collections with different numbers of isolates; estimates of *nENDI* belong to the [0; 1] interval.

Untypical *P. triticina* races were determined using the metric of individual singularity (Equations (1)–(5) in [18]). The singularity of each race was calculated based on the *sm* dissimilarity of that focus pathotype from all other pathotypes in a collection.

The effective number of different *P. triticina* collections (*ENDC*) was calculated according to [19] as the ^1^*D*(*ADW_KB_*) metric based on the $ADW={ADW}_{KB}$ dispersion (Average *Distance* between collections Within a given set of collections) with regard to the *KB* distance between collections (only polymorphic virulence loci were included). Values of *ENDC* range from 1, if all collections are identical, to an actual number of collections when they are absolutely different.

UPGMA dendrograms of relationships among the races in all *P. triticina* collections with regard to the simple mismatch dissimilarity between them was generated using the SAHN program of the NTSYSpc package, version 2.2 (Exeter Software, Setauket, NY, USA). The same software was used to construct the UPGMA dendrogram of relationships among the regional *P. triticina* collections and groups with regard to the *KB* distance between them. Other calculations were performed with VIRULENCE ANALYSIS TOOL (VAT) software [20] (https://en-lifesci.tau.ac.il/profile/kosman/vat, accessed on 1 August 2026) and FUNCTIONAL DIVERSITY ANALYSIS TOOLS (FDAT) software (https://en-lifesci.tau.ac.il/kosman/fdat, accessed on 1 August 2026). Both packages are available at https://en-lifesci.tau.ac.il/profile/kosman (accessed on 1 August 2026).

## 3. Results

### 3.1. Virulence Phenotypes of P. triticina on KASIB-26 Accessions

A total of 195 single-pustule isolates, derived from spring wheat accessions evaluated within the Kazakhstan–Siberia Network (KASIB-26) in 2025, were analyzed (34 from Chelyabinsk, 24 from Kurgan, 52 from Tyumen, 43 from Omsk, and 42 from Kazakhstan). Isolates obtained from lines Lutescens 22144 and 434/sp2/21 were present in all five regions. On line Lutescens 22144, the predominant race TCTTR was observed in Chelyabinsk, Kurgan, Tyumen, and Kazakhstan (Table 2); in Omsk, however, race MCTKH was detected on this line, which differed from TCTTR by avirulence to *Lr2a*, *Lr2b*, *Lr2c*, and *Lr15*. A greater phenotypic variability was noted on line 434/sp2/21 (four races): TCTTR was dominant and found in Chelyabinsk, Kazakhstan and Omsk; TFTTR, distinguished by avirulence to *Lr24*, was identified in Kurgan; QCTTR, characterized by avirulence to *Lr2a,* was found in Tyumen; and in Omsk, the second race—MCTKG—was detected on this line (in addition to TCTTR). Phenotypic variation in *P. triticina* was also evident across all other spring wheat lines studied.

### 3.2. Regional Composition and Relationships of P. triticina Virulence Phenotypes

**Figure 2:**
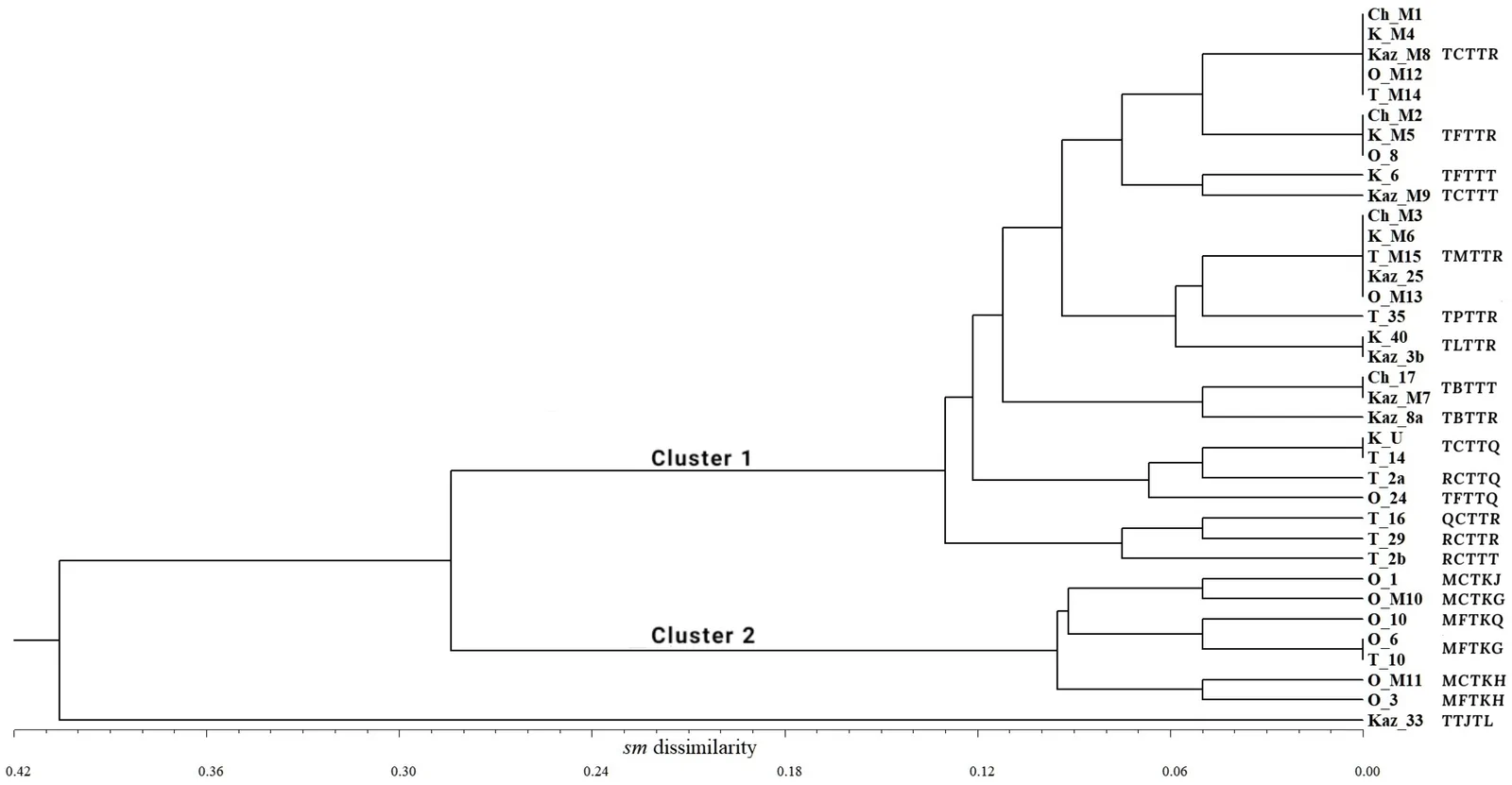
UPGMA dendrogram of relationships among all 22 virulence phenotypes of *P. triticina* identified in the KASIB-26 collections of isolates. Region abbreviations: Ch—Chelyabinsk, K—Kurgan, T—Tyumen, O—Omsk, Kaz—North Kazakhstan. The phenotype designations include the corresponding region and accession number; for example, O_1 means phenotype from Omsk collected in wheat accession No. 1 according to Table 1, Ch_M1 means phenotype from Chelyabinsk collected in wheat accessions that belong to group M1.

The UPGMA dendrogram generated with the simple mismatch dissimilarities demonstrated relationships of virulence phenotypes in all regions (Figure 2). The first cluster (Cluster 1) included 15 races that comprised 88% of isolates from all five regions, whereas the second one (Cluster 2) was much smaller, with only 6 races from Omsk and Tyumen (24 and 2 isolates, respectively). One Kazakh phenotype (Kaz_33) was strongly differentiated from all other phenotypes. The Omsk collection was clearly divided into two groups: the first group (O_G1) comprised only M-race isolates, whereas the second group (O_G2) was composed of more virulent T-race isolates. Although almost all Tyumen isolates were assigned to Cluster 1 (except T_10), they still displayed internal differentiation into two groups, T_G1 and T_G2 (see the UPGMA dendrogram in Figure S1 and further analyses).

The group composition of wheat accessions is described according to their order in the UPGMA from the top to bottom:

Ch_M1 includes isolates from wheat accessions No. 1, 6, 7, 15, 16, 18, 29, 31 (Table 1);
K_M4—No. 1, 15, 32, 48;
Kaz_M8—No. 1, 2, 3b, 5, 11b, 12a, 13–17, 22, 24a, 32, 40;
O_M12—No. 5a, 9b, 16b, 17, 28, 47;
T_M14—No. 3–8, 11–13, 15, 17, 24, 26, 27, 30, 32, 36;
Ch_M2—No. 2, 4, 10, 11, 36, 48;
K_M5—No. 14, 16, 41;
Kaz_M9—from No. 9, 11a, 12b, 18, 24b;
Ch_M3—No. 30, 37;
K_M6—No. 18, 24;
O_M13—No. 22, 26; Kaz_M7—No. 8b, 26;
O_M10—No. 7, 9a, 12, 16a;
O_M11—No. 2, 5bc, 15, 29, 32;
T_M15—No. 22, 28, 37.

The composition and frequency of races in each regional population and its respective subgroups are summarized in Table 3. In total, 22 races were identified among the 195 isolates examined. Races TCTTR and TMTTR were present across all regions. Race TFTTR was observed at three Russian sites—Chelyabinsk, Kurgan, and Omsk—while races TBTTT, TCTTQ, TLTTR, and MFTKG were detected in two different regions. The remaining races were each confined to a single region. Average virulence complexity values were approximately similar among isolates from Chelyabinsk, Kurgan, and Kazakhstan (RVC = 0.81–0.83), but were lower in the Tyumen and Omsk collections (0.76 and 0.69, respectively). The range of RVC for individual isolates was much higher in the Omsk (0.55–0.85) and Tyumen (0.6–0.9) collections. The two groups of *P. triticina* isolates within each Omsk (O_G1 and O_G2) and Tyumen (T_G1 and T_G2) collection differed considerably by their average RVC.

The most unique races among all 22 detected in the whole KASIB-26 collection are shown in Table 4. The TTJTL race identified on line Erythrospermum 235-21 in Kazakhstan (Table 2) was of the highest singularity (13.98). This race was of high virulence complexity (RVC = 0.8) and exhibited a very specific virulence profile, with rare virulences to *Lr9* and *Lr24* and being the only race avirulent to *Lr3ka*, *Lr18*, and *Lr30*. All other highly singular races were identified in the Omsk and Tyumen collections, belonged to Cluster 2 (Figure 2) and displayed relatively low virulence complexity (0.55–0.65).

### 3.3. Virulence Frequency in Regional P. triticina Collections

Most of the *P. triticina* isolates tested exhibited virulence to *Lr1*, *Lr3a*, *Lr3bg*, *Lr3ka*, *Lr11*, *Lr14a*, *Lr14b*, *Lr17*, *Lr18*, *Lr26* and *Lr30*, with frequencies ranging from 88.1% to 100% (Table 5). Virulence to *Lr16* was detected only in Kazakhstan (two isolates of the race TTJTL). Virulence frequencies on lines carrying *Lr2a*, *Lr2b*, *Lr2c* and *Lr15* were significantly lower in Omsk (44.2–48.8%) compared with all other regions (96.2–100%). Most isolates avirulent to *Lr2a*, *Lr2b* and *Lr2c* were also avirulent to *Lr15*, except for two isolates from Omsk. Virulence frequencies for *Lr9* varied from 9.3% (Omsk) to 25.0% (Kurgan), for *Lr19* from 1.9% (Tyumen) to 28.6% (Kazakhstan)*,* and for *Lr24* from 4.8% (Kazakhstan) to 35.3% (Chelyabinsk). The Tyumen isolate groups (T_G1 and T_G2) exhibited significant differences in virulence to *Lr9* and *Lr24*, with T_G2 displaying higher virulence frequencies (Table 5). Similarly, the Omsk groups (O_G1 and O_G2) differed markedly in their virulence frequencies to *Lr2a*, *Lr2b*, *Lr2c*, *Lr9*, *Lr15*, and *Lr20*, with O_G2 showing larger values; in contrast, virulence to *Lr19* was more prevalent in O_G1.

### 3.4. Variability Within and Among Regional/Group P. triticina Collections

Analysis of *P. triticina* virulence phenotypes within each separate region revealed one untypical race in each Tyumen (T_10, MFTKG) and Kazakhstan (Kaz_33, TTJTL) collection. In addition, a putative division into two groups of all other isolates in Tyumen (T_G1 and T_G2) as well as in Omsk (O_G1 and O_G2) seemed very probable. Therefore, we estimated variability within regions and groups and tested differentiation among them.

Based on the integral *nENDI* metric and *KW*-dispersion, the most significant variable was the O_G1 group of isolates in the Omsk region (*nENDI* = 0.077, *KW* = 0.092; Table 6), though the simple richness was larger in the Kurgan collection (0.217). The least significant variable was the T_G1 group of isolates in the Tyumen region (*nENDI* = 0.017, *KW* = 0.029), though the simple richness was smaller in the Chelyabinsk collection (0.091). Note, however, that variability within all regions/groups considered separately (Table 6) was very low (*nENDI* maximum 0.077 was much smaller than the theoretically possible 1), which is evidence of their homogeneity.

**Figure 3:**
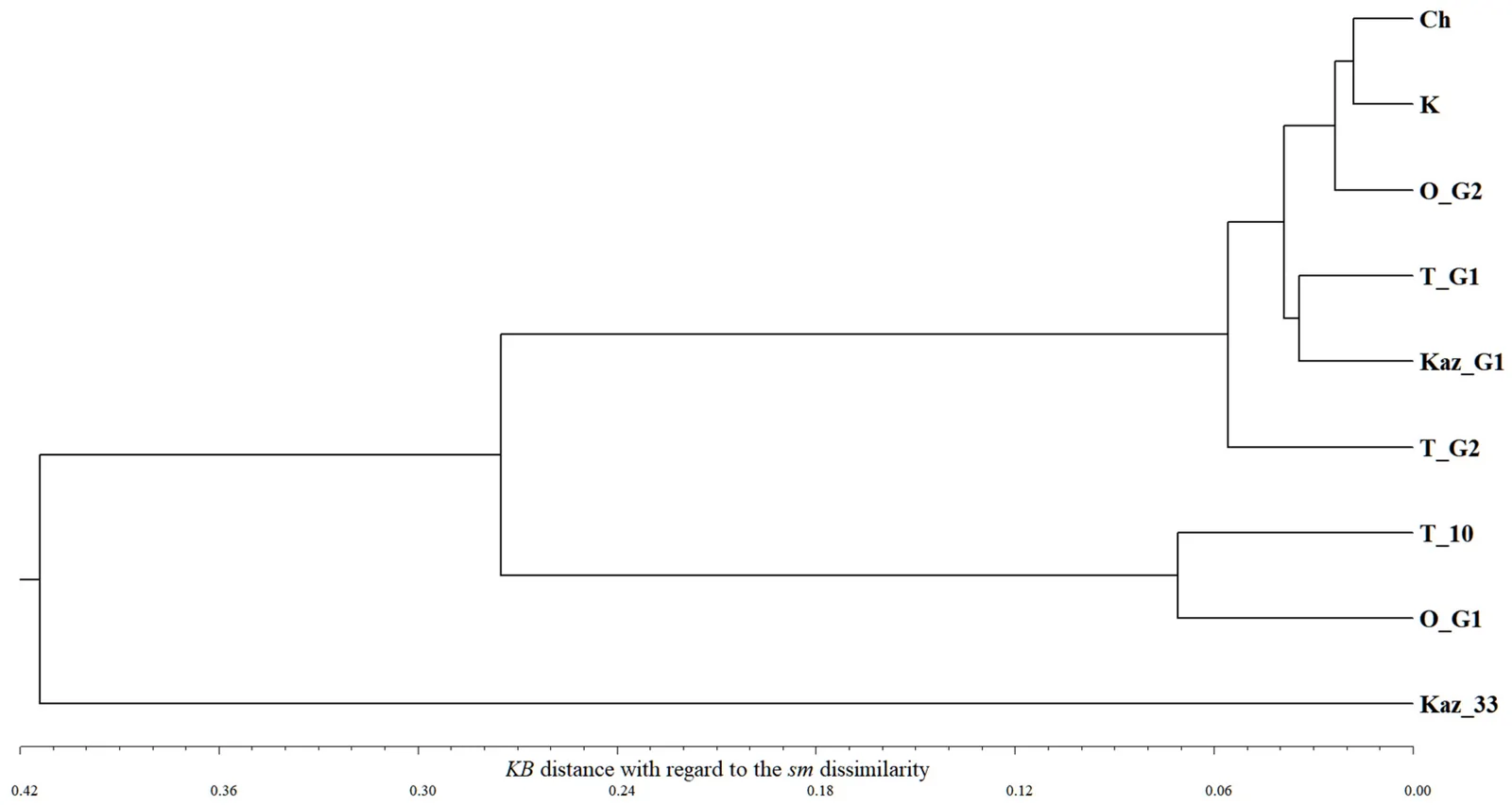
UPGMA dendrogram of relationships among regional/group collections of *P. triticina* isolates in 2025. Region/group abbreviations: Ch—Chelyabinsk, K—Kurgan, T—Tyumen, O—Omsk, Kaz—North Kazakhstan; T_G1—group 1 in Tyumen, etc.

It was proven that two groups of Tyumen isolates, T_G1 and T_G2, were significantly different from each other (*p* < 0.01; Table 7), as were two groups of Omsk isolates, O_G1 and O_G2. This means that the virulence structure of the *P. triticina* population in the Omsk and Tyumen regions was more complex than in other studied regions (see also Figure 3). Moreover, both the Omsk pathogen group O_G1 and the Tyumen pathogen group T_G2 were significantly different from all other regional/group collections (*p* < 0.01; Table 7), whereas the former one was the most distinct *P. triticina* group, with the *KB* distance being in a range from 0.228 to 0.275 between O_G1 and each other collection.

The atypical isolate T_10 from Tyumen (race MFTKG) clustered closely with the O_G1 group of Omsk isolates, and together they formed a separate cluster that was clearly differentiated from all other groups in the UPGMA dendrogram (Figure 3). As expected, the group of isolates from Kazakhstan with the most singular virulence phenotype, Kaz_33 (race TTJTL), is located far apart from all other groups on the dendrogram. On the other hand, five groups of *P. triticina* isolates from Chelyabinsk (Ch), Kurgan (K), Omsk (O_G2), Tyumen (T_G1) and Kazakhstan (Kaz_G1) were very closely related (Figure 3) and statistically indistinguishable by the differentiation test (*p* > 0.05). The pairwise *KB* distances between those five groups were very small (ranging from 0.018 to 0.047; Table 7), as was the effective number of different collections, *ENDC* = 1.131 (theoretically, in this case of five groups, *ENDC* values belong to the [1,5] interval). The obtained estimate was very close to 1, indicating the existence of a single *P. triticina* collection that combines the five groups from different regions.

## 4. Discussion

In the present study, we characterized *P. triticina* isolates collected from spring wheat accessions evaluated within the Kazakhstan–Siberia Network (KASIB-26) in the Ural and Western Siberian parts of Russia and northern Kazakhstan. The geographic distances from the easternmost sampling site in the Chelyabinsk region (located at the Europe–Asia boundary) were approximately 250 km to Kurgan, 350 km to Tyumen, 750 km to Omsk, and 850 km to the collection site in Akmola Oblast, northern Kazakhstan. The distance between the two Western Siberian locations, Tyumen and Omsk, was approximately 550 km. Spring wheat is the predominant crop across all these areas. Among wheat rust diseases, leaf rust is the most common and occurs annually with varying degrees of severity [21,22].

Long-term virulence monitoring of Russian *P. triticina* populations, carried out at the All-Russian Institute of Plant Protection (Saint Petersburg) continuously since 1990, has demonstrated the presence of two distinct population groups across Russia: European and Asian. The western group encompassed populations from the Northwest, Central European Russia, and the North Caucasus, whereas the eastern group comprised populations from the Urals and Western Siberia. The wheat-growing regions along the Volga River—the easternmost in the European part of Russia—have been characterized by a mix of the European and Asian *P. triticina* populations because typical pathogen races from both of them coexisted there. The primary distinction between the European and Asian pathogen populations was expressed by significantly lower virulences to *Lr2a*, *Lr2b*, *Lr2c* and *Lr15* in the European regions compared to the Asian ones. Additionally, the Asian *P. triticina* populations were characterized by high virulence frequencies to *Lr9* [6.7].

For the wheat leaf rust pathogen, conventional virulence analysis continues to be the most widely adopted and practically relevant approach worldwide [23,24,25,26,27]. It allows for the identification of actual changes in pathogen virulence, which is particularly important for practical breeding. At the Cereal Rust Diseases Laboratory (USDA) and other major international reference laboratories, molecular markers are primarily utilized for global-scale population studies, while annual pathogen surveillance—comparable to our monitoring—relies on virulence phenotyping. Molecular markers can effectively improve the theoretical depth and practical value of phytopathological research. However, they are predominantly used in global analyses [28,29,30,31,32,33,34]. Both virulence and microsatellite markers were employed in our previous analogous study on Asian populations in some of these regions in 2016 [6], as well as on Russian regional populations overall.

In the current study, we performed a more detailed characterization of the population structure of *P. triticina* with respect to virulence in the most western Asian regions of Russia and in the neighboring territories of Kazakhstan, employing a common set of wheat cultivars as inoculum sources. Using a set of 20 differentials (Thatcher near-isogenic lines), 22 races were identified among the 195 isolates examined. Races TCTTR and TMTTR were present across all regions, while races TFTTR, TCTTQ, TLTTR, TBTTT, and MFTKG were detected in two or more regions. Races TCTTR and TCTTQ belong to a group widely distributed in the Asian regions and have been detected annually since 2001 [6,7,8,9]. Race TLTTR, which differs from these races by virulence to *Lr9,* has been regularly recorded in the region since 2010, when the effectiveness of *Lr9* was overcome in the Asian regions of Russia and Kazakhstan. Until 2025, all isolates virulent to *Lr9* were avirulent to *Lr26*, suggesting that varieties carrying both genes would be resistant to the pathogen. In the present study, however, race TMTTR, virulent to both genes, was recorded at all locations for the first time. Races MCTKH and MCTKG, detected in the current analysis only in the Omsk collection, have also been regularly observed in other Western Siberian, Ural, and Kazakh populations in previous years, though their frequencies were substantially lower than those of the T-group races. Races characterized by virulence to *Lr19* (TCTTT, TBTTT), recorded in the current analysis in the Chelyabinsk and northern Kazakhstan collections, were more prevalent in the region before 2010 and have occurred only sporadically since then.

Several new *P. triticina* races virulent to *Lr24* were identified in the present study. They belong to the -M---, -F---, -P---, and -T--- race groups (defined based on the second set of four differentials), all virulent to Tc*Lr24*. Note that the only such race—TFTTR, from the -F--- group—identified in the present study and virulent to the previously effective gene, *Lr24*, was previously recorded in the Chelyabinsk region in 2024, and by 2025, it had already been detected in Omsk and Kurgan. Single-step mutations can explain the origin of virulence to *Lr24* from TCTTR. Moreover, a notably singular race, TTJTL, was recovered from Kazakhstan in the current survey; this race is virulent to both *Lr9* and *Lr24* and is distinguished by its rare avirulences to *Lr3ka*, *Lr18* and *Lr30*. Spring wheat varieties carrying the *Lr24* gene have been cultivated in the studied territory since 2020 (cultivars Leader 80 and Aina in Russia and Kazakhstan, respectively). Leaf rust on these previously highly resistant varieties has been observed since 2024. The emergence of new virulence phenotypes and the changes in distribution and abundance of previously widespread phenotypes indicate that the Asian *P. triticina* population is highly dynamic.

The findings of this study indicate that the *Lr* genes currently employed have low potential for use in breeding for leaf rust resistance in Russia and Kazakhstan. None of the 20 *Lr*-genes represented in the differential lines employed in this study proved to be highly effective. Virulence frequencies to the majority of *Lr*-genes, except *Lr24*, were consistent with those reported in earlier investigations [4,5,6,7]. Virulence to *Lr24* is widely distributed across the North American continent and Australia [12,35]. From the mid-2010s onwards, it has also been reported in Western Europe [36]. In Russia, isolates carrying virulence to *Lr24* were first recorded in the Northwest and the Southern Ural regions in 2024 [7]. In the present study, isolates exhibiting virulence to *Lr24* were identified not only on the cultivar Inzhu, which harbors this resistance gene, but also on wheat accessions that do not carry it. In Russia and Kazakhstan, the combination of *Lr9* and *Lr26* was previously effective against leaf rust, despite both genes being ineffective when deployed separately. This effectiveness was attributable to the absence of isolates with combined virulence to both genes in the local populations. However, in 2025, we first observed leaf rust infection of accessions protected by both genes: *P. triticina* isolates originating from line Lutescens 14/10-11 belonged to race TMTTR, which is virulent to both *Lr9* and *Lr26* genes, though this line possesses both of those resistance genes. No virulence to *Lr16* was identified among the Russian *P. triticina* isolates analyzed in this study, and only sporadic virulence was recorded in the Kazakhstan collection. Although the *Lr16*-carrying Thatcher line displays a 1–2 infection type at the seedling stage, it exhibits moderate susceptibility with an X-type reaction under field conditions. Consequently, *Lr16* is not a promising candidate for breeding programs targeting leaf rust resistance.

Isolates recovered from lines Lutescens 22144 and 434/sp2/21 were represented in all five regions. Molecular marker analysis demonstrated that line Lutescens 22144 harbors the ineffective *Lr3* gene, whereas line 434/sp2/21 contains the *Lr26* gene. On line Lutescens 22144, the predominant race TCTTR was recorded in four regions (Chelyabinsk, Kurgan, Tyumen and Kazakhstan), whereas the less virulent race MCTKH was detected in Omsk. Line 434/sp2/21 exhibited greater phenotypic diversity of the pathogen: the common race TCTTR was present in three regions—Chelyabinsk, Kazakhstan and Omsk; in Omsk, an additional race—MCTKG—was also identified; race TFTTR was observed in Kurgan; and race QCTTR was found in Tyumen. Line L-3185 showed high field resistance, with only single pustules (1% severity) in Chelyabinsk and Tyumen. Molecular markers identified the ineffective genes *Lr3* and *Lr26* in this line. Race TCTTR was prevalent on line L-3185 in Tyumen, whereas race TMTTR was detected in Chelyabinsk. Overall, our results show both similarities and differences in pathogen population structure on the same wheat genotypes across distant locations.

High similarity was observed between *P. triticina* collections from Chelyabinsk, Kurgan, almost all isolates from Kazakhstan (Kaz_G1), the Tyumen group T_G1, and the Omsk group O_G2. Over 80% of the studied isolates belonged to this combined set, and many of their phenotypes were traditional races widely distributed in Asian *P. triticina* populations. A considerably different group of isolates from the Omsk region (group O_G1) was characterized by avirulence to the *Lr2a*, *Lr2b*, *Lr2c*, and *Lr15* genes. High variability of another wheat pathogen, *Puccinia graminis*, in the Omsk region was also documented in study of stem rust [37]. On the basis of virulence phenotypes, two distinct *P. graminis* populations were identified in the Omsk region during 2017–2018. It has been hypothesized that this differentiation may be associated with the respective origins of those populations from asexual and sexual reproductive cycles.

Presence of M-races in *P. triticina* populations in Asian regions of Russia showed significant year-to-year variation. In earlier surveys conducted between 2013 and 2016, M-race isolates were not detected in samples either from Omsk or from Chelyabinsk [5,6,38,39,40], nor in the Kurgan region in 2016 [5]. In 2017, a comparable analysis of *P. triticina* isolates from spring wheat accessions studied within the KASIB-18 program identified M-races in the Chelyabinsk and Omsk collections, but not in North Kazakhstan [5]. The frequency of M-race isolates was substantially higher in Chelyabinsk than in Omsk (30% vs. 5%). Subsequent annual monitoring of *P. triticina* in the Chelyabinsk region from 2021 to 2024 revealed isolates of M-races each year except 2022, with frequencies varying from 2.8% in 2024 to 23% in 2021 and 2023. In this study, MCTKH and MCTKG were predominant M-races in the Omsk collection in 2025, and they were also observed in the Chelyabinsk region, with isolate frequencies of 3.8% and 11.5% in 2021, and 11.5% and 7.7% in 2023, respectively [41].

Detailed studies on the epidemiology of *P. triticina* in Western Siberia and Northern Kazakhstan were carried out by B.G. Reiter in the 1960s–1980s [42,43]. It was shown that pathogen infection can be local and persist on wild grasses, as well as be introduced from other regions. In addition, alternate host plants (*Thalictrum* sp., *Isopyrum fumarioides*) are widespread in the region. Long-term studies of virulence and race composition of the pathogen revealed high virulence of local *P. triticina* populations and their high degree of variability towards increased virulence. It was suggested that this may be caused by extreme overwintering conditions, which are survived by the most locally adapted clones, whereas clones introduced from other regions die. Long-term observations of airborne spore load at different locations in the region showed that aerial introduction of infection into the Asian part of Russia and northern Kazakhstan is possible from the southern or southwestern regions of the European part of Russia, as well as from the Middle Volga region and western areas of Kazakhstan [43]. Mass spread of introduced infection is substantially limited by the hydrothermal conditions of different subzones of the region (foothills, forest steppe, subtaĭga, and steppe zones). It was shown that when infection is introduced from other regions, the composition of local Asian *P. triticina* populations changes significantly. Along with traditional highly virulent races, races characteristic of European and North Caucasian populations are widely represented, which is noted in the present analysis for Omsk *P. triticina* samples, where races MCTKG and MCTKH, typical of the European part of Russia, were widely represented [6,7].

The comprehensive analysis of *P. triticina* samples collected in the Ural and Western Siberian regions of Russia and northern Kazakhstan indicates their heterogeneity. Although in 2025 distinct pathogen groups were identified within the Omsk and Tyumen regions and the untypical race was found in Kazakhstan, the overall population structure remained stable during the last decade, with a predominant complex of highly virulent races persisting across the whole territory. Alongside traditional races, new races with previously absent virulences were detected this year, probably influenced by the cultivation of various genetically protected cultivars. Our outcomes emphasize again that annual monitoring of *P. triticina* is absolutely necessary for controlling leaf rust disease of wheat because regular observations enable tracking structural changes in the virulence of the pathogen and the emergence of new races. The long-term population study allows for a more comprehensive and accurate characterization of regional dynamics of *P. triticina* and facilitates incorporation of the obtained knowledge into breeding programs aimed at developing resistant cultivars.

## Figures and Tables

**Table 1:** Field of leaf rust severity (%) in accessions of spring wheat from which *P. triticina* isolates were collected.

Accession		Disease Severity (%)				Origin of Isolates ^b^
No ^a^		Ch ^b^	K	T	O	
1	Aktyubinskaya 70	100	100	80	100	Ch, O, K, Kaz
2	Line 327/22-KSm	100	100	80	100	Ch, T, O, Kaz
3	Line 342/22KSm	100	100	80	100	T, O, Kaz
4	Line 241/14	100	100	80	100	T, Ch
5	Line 169/15	90	60	80	60	O, T, K, Kaz
6	Line 249/14-4	100	100	80	100	Ch, T, K, O
7	Line 13/94-04-1	100	100	80	100	Ch, T, O
8	Line 261-10-5	100	100	80	100	T, O, Kaz
9	Line 24/02-10-4	100	100	50	100	O, Kaz
10	Inzhu	100	80	50	100	Ch, T, O
11	QS 2134	100	80	50	100	Ch, T, Kaz
12	Lutescens 48 140/11	100	100	60	100	T, O, Kaz
13	Lutescens 22102	100	100	80	100	T, Kaz
14	Lutescens 22138	100	100	80	100	T, Kaz
15	Lutescens 22144	100	100	70	100	Ch, K, T, O, Kaz
16	434/sp2/21	100	80	50	100	Ch, K, T, O, Kaz
17	658 sp2/21	90	60	10	5	Ch, T, O, Kaz
18	638 sp2/20	90	60	20	40	Ch, K, Kaz
22	Pamyati Azieva	90	80	80	100	T, O, Kaz
24	Astana 2	100	70	80	100	K, T, O, Kaz
25	Omskaya 35	100	100	70	100	Kaz
26	Saratovskaya 29	100	70	80	100	T, O, Kaz
27	L-Cht-464	90	30	60	5	T, O
28	L-Gb-28-16	100	80	100	100	T, O
29	L-Dc-2/18	50 R	30	60	10	Ch, T, O
30	L-3185	1	0	1	0	Ch, T
31	KS 112/08-2	5	0	5	5	Ch
32	KS 285/12-1674	80	15	80	80	K, T, O, Kaz
33	Erythrospermum 235-21	100	30	80	100	Kaz
35	Omskiy ametist	90	30	80	100	T
36	Lutescens 241/13-1	90	20	80	100	Ch, T
37	Lutescens 14/10-11	90	5	60	0	Ch, T
40	Л 2142	90	30	100	100	K, Kaz
47	PYAM/SM, ZH-22-60	100	70	100	100	O
48	KS 29/17y	90	30	80	100	Ch

^a^ Accession number in the KASIB-26 list; ^b^ region abbreviations: Ch—Chelyabinsk, K—Kurgan, T—Tyumen, O—Omsk, Kaz—North Kazakhstan.

**Table 2:** Races of *P. triticina* identified in different regions on wheat accessions studied in the Kazakhstan–Siberia Network in 2025 (KASIB-26).

No ^a^	Accession	Region				
		Ch ^b^	K	T	O	Kaz
1	Aktyubinskaya 70	TCTTR ^c^	TCTTR	-	MCTKJ	TCTTR
2	Line 327/22-KSm	TFTTR	-	RCTTQ, RCTTT	MCTKH	TCTTR
3	Line 342/22KSm	-	-	TCTTR	MFTKH	TCTTR, TLTTR
4	Line 241/14	TFTTR	-	TCTTR	-	-
5	Line 169/15	-	-	TCTTR	TCTTR, MCTKH	-
6	Line 249/14-4	TCTTR	TFTTT	TCTTR	MFTKG	-
7	Line 13/94-04-1	TCTTR	-	TCTTR	MCTKG	-
8	Line 261-10-5	-	-	TCTTR	TFTTR	TBTTR, TBTTT
9	Line 24/02-10-4	-	-	-	MCTKG, TCTTR	TCTTT
10	Inzhu	TFTTR	-	MFTKG	MFTKQ	-
11	QS 2134	TFTTR	-	TCTTR	-	TCTTT, TCTTR
12	Lutescens 48 140/11	-	-	TCTTR	MCTKG	TCTTR, TCTTT
13	Lutescens 22102	-	-	TCTTR	-	TCTTR
14	Lutescens 22138	-	-	TCTTQ	-	TCTTR
15	Lutescens 22144	TCTTR	TCTTR	TCTTR	MCTKH	TCTTR
16	434/sp2/21	TCTTR	TFTTR	QCTTR	MCTKG, TCTTR	TCTTR
17	658 sp2/21	TBTTT		TCTTR	TCTTR	TCTTR
18	638 sp2/20	TCTTR	-	-	-	TCTTT
22	Pamyati Azieva	-	-	TMTTR	TMTTR	TCTTR
24	Astana 2	-	TMTTR	TCTTR	TFTTQ	TCTTR, TCTTT
25	Omskaya 35	-	-	-	-	TMTTR
26	Saratovskaya 29	-	-	TCTTR	TMTTR	TBTTT
27	L-Cht-464	-	-	TCTTR	TCTTR	-
28	L-Gb-28-16	-	-	TCTTR	TCTTR	-
29	L-Dc-2/18	TCTTR	-	RCTTR	MCTKH	-
30	L-3185	TMTTR	-	TCTTR	-	-
31	KS 112/08-2	TCTTR	-	-	-	-
32	KS 285/12-1674	-	TCTTR	TCTTR	MCTKH	TCTTR
33	Erythrospermum 235-21	-	-	-	-	TTJTL
35	Omskiy ametist	-	-	TPTTR	-	-
36	Lutescens 241/13-1	TFTTR	-	TCTTR	-	-
37	Lutescens 14/10-11	TMTTR	-	TMTTR	-	-
40	Л 2142	-	TLTTR	-	-	TCTTT
47	PYAM/SM, ZH-22-60	-	-	-	TCTTR	-
48	KS 29/17y	TFTTR	-	-	-	-

^a^ Accession number in the KASIB-26 list; ^b^ region abbreviations: Ch—Chelyabinsk, K—Kurgan, T—Tyumen, O—Omsk, Kaz—North Kazakhstan; ^c^ the hexadecimal code of race designation was determined according to Long and Kolmer [11].

**Table 3:** Race composition of *P. triticina* in regional collections in 2025.

Race ^a^	Avirulence to Thatcher with *Lr* Genes		Frequency of Races Across Regions/Groups ^b^								
		RVC ^c^	Ch	K	T	T_G1	T_G2	O	O_G1	O_G2	Kaz
TBTTR	9, 16, 19, 24, 26	0.75									2.4
TBTTT	9, 16, 24, 26	0.8	5.9								7.1
TCTTQ	9, 16, 19, 20, 24	0.75		4.2	3.8	4.8					
TCTTR	9, 16, 19, 24	0.8	47.1	37.5	65.4	81		25.6		57.9	61.9
TCTTT	9, 16, 24	0.85									16.7
TFTTQ	9, 16, 19, 20	0.8						4.7		10.5	
TFTTR	9, 16, tri 19	0.85	35.3	25				4.7		10.5	
TFTTT	9, 16	0.9		8.3							
TLTTR	16, 19, 24, 26	0.8		8.3							2.4
TMTTR	16, 19, 24	0.85	11.8	16.7	11.5		75	9.3		21.1	4.8
TPTTR	16, 19	0.9			3.8		25				
TTJTL	3ka, 18, 19, 20, 30	0.8									4.8
RCTTT	2c, 9, 16, 24	0.8			1.9	2.4					
RCTTR	2c, 9, 16, 19, 24	0.75			3.8	4.8					
RCTTQ	2c, 9, 16, 19, 20, 24	0.7			1.9	2.4					
QCTTR	2c, 3a, 9, 16, 19, 24	0.7			3.8	4.8					
MFTKQ	2a, 2b, 2c, 9, 16, 19, 20	0.65						4.7	8.3		
MFTKH	2a, 2b, 2c, 9, 15, 16, 19	0.65						4.7	8.3		
MFTKG	2a, 2b, 2c, 9, 15, 16, 19, 20	0.6			3.8			4.7	8.3		
MCTKJ	2a, 2b, 2c, 9, 15, 16, 20, 24	0.6						4.7	8.3		
MCTKH	2a, 2b, 2c, 9, 15, 16, 19, 24	0.6						23.3	41.8		
MCTKG	2a,2b,2c,9, 15, 16, 19, 20, 24	0.55						14	25		
Isolates ^d^			34	24	52	42	8	43	24	19	42
races ^d^			4	6	9	6	2	10	6	4	7
mean RVC ^e^			0.82	0.83	0.76	0.79	0.86	0.69	0.6	0.82	0.81
min RVC ^e^			0.8	0.8	0.6	0.7	0.85	0.55	0.55	0.8	0.75
max RVC ^e^			0.85	0.9	0.9	0.8	0.9	0.85	0.65	0.85	0.85

^a^ Hexadecimal code of race designation. The differential host panel was composed of five groups, each comprising four near-isogenic lines carrying individual *Lr* resistance genes. Set 1: *Lr1*, *Lr2a*, *Lr2c*, and *Lr3*; set 2: *Lr9*, *Lr16*, *Lr24*, and *Lr26*; set 3: *Lr3ka*, *Lr11*, *Lr17,* and *Lr30*; set 4: *Lr2b*, *Lr3bg*, *Lr14a*, and *Lr14b*; set 5: *Lr15*, *Lr18*, *Lr19*, and *Lr20*. ^b^ Region/group abbreviations: Ch—Chelyabinsk, K—Kurgan, T—Tyumen, O—Omsk, Kaz—North Kazakhstan, T_G1—group 1 in Tyumen, etc.; ^c^ RVC—relative virulence complexity; ^d^ number of isolates and races in the corresponding region/group; ^e^ regional and group RVC estimates for isolates.

**Table 4:** Singularity of *P. triticina* virulence phenotypes in the KASIB-26 collection.

Race Name ^a^	Race ^b^	Singularity	Region/Group ^c^	RVC ^d^
Kaz_33	TTJTL	13.98	Kaz	0.8
O_1	MCTKJ	9.23	O_G1	0.6
O_6	MFTKG	8.44	O_G1	0.6
T_10	MFTKG	8.44	T	0.6
O_3	MFTKH	7.97	O_G1	0.65
O_M10	MCTKG	7.93	O_G1	0.55
O_10	MFTKQ	7.69	O_G1	0.65
O_M11	MCTKH	7.35	O_G1	0.6

^a^ Race names include the corresponding region and accession number; for example, Kaz_33 means a phenotype from Kazakhstan collected in wheat accession No. 33 according to Table 1, O_M10 means a phenotype from Omsk collected in wheat accessions that belong to group M10 (see Figure 2), etc.; ^b^ hexadecimal code of race designation; ^c^ region/group abbreviations: T—Tyumen, O—Omsk, Kaz—North Kazakhstan; ^d^ RVC—relative virulence complexity.

**Table 5:** Virulence frequency (%) of *P. triticina* isolates on the Thatcher lines in the regional collections in 2025.

*Lr* Genes	Frequency of Isolates Across Russia and Kazakhstan								
	Ch ^a^	K	T	T_G1	T_G2	O	O_G1	O_G2	Kaz
*Lr1*	100	100	100	100	100	100	100	100	100
*Lr2a*	100	100	96.2	100	100	44.2	0	100	100
*Lr2b*	100	100	96.2	100	100	44.2	0	100	100
*Lr2c*	100	100	84.6	85.7	100	44.2	0	100	100
*Lr3a*	100	100	96.2	100	95.2	100	100	100	100
*Lr3bg*	100	100	100	100	100	100	100	100	100
*Lr3ka*	100	100	100	100	100	100	100	100	95.2
*Lr9*	11.8	25	15.4	0	100	9.3	0	21.1	11.9
*Lr11*	100	100	100	100	100	100	100	100	100
*Lr14a*	100	100	100	100	100	100	100	100	100
*Lr14b*	100	100	100	100	100	100	100	100	100
*Lr15*	100	100	96.2	100	100	48.8	8.3	100	100
*Lr16*	0	0	0	0	0	0	0	0	4.8
*Lr17*	100	100	100	100	100	100	100	100	100
*Lr18*	100	100	100	100	100	100	100	100	95.2
*Lr19*	5.9	8.3	1.9	2.4	0	4.7	8.3	0	26.2
*Lr20*	100	95.8	90.6	92.9	100	67.4	50	89.5	95.2
*Lr24*	35.3	33.3	7.7	0	25	23.3	25	21.1	4.8
*Lr26*	94.1	91.7	100	100	100	100	100	100	88.1
*Lr30*	100	100	100	100	100	100	100	100	95.2

^a^ Region/group abbreviation: Ch—Chelyabinsk, K—Kurgan, T—Tyumen, O—Omsk, Kaz—North Kazakhstan; T_G1—group 1 in Tyumen, etc.

**Table 6:** Variability within *P. triticina* regional/group collections in 2025.

Region/Group ^a^	*NI* ^b^	*NR* ^c^	Richness ^d^	*KW* ^e^	*ENDI* ^f^	*nENDI* ^g^
Ch	34	4	0.091	0.059	2.51	0.046
K	24	6	0.217	0.079	2.54	0.067
Kaz_G1	40	6	0.128	0.045	2.26	0.032
O_G1	24	5	0.174	0.092	2.78	0.077
O_G2	19	4	0.167	0.053	1.73	0.041
T_G1	42	6	0.122	0.029	1.68	0.017
T_G2	8	2	0.143	0.053	1.25	0.036

^a^ Region/group abbreviation: Ch—Chelyabinsk, K—Kurgan, T—Tyumen, O—Omsk, Kaz—North Kazakhstan, T_G1—group 1 in Tyumen, etc.; ^b^ *NI*—number of isolates in each collection; ^c^ *NR*—number of races in each collection; ^d^ Richness—simple richness as the number of races per isolate normalized to the [0; 1] interval: $\left(NR-1\right)/\left(NI-1\right)$; ^e^ *KW*—assignment-based dispersion within the collection; ^f^ effective number of different isolates in each collection, 1≤ENDI≤NI; ^g^ normalized effective number of different isolates in each collection, aimed at comparison samples of different size (number of isolates): $\left(ENDI-1\right)/\left(NI-1\right)$, 0≤nENDI≤1.

**Table 7:** *KB* distance (above diagonal) and significance of pairwise differentiation (below diagonal) between *P. triticina* regional collections in 2025.

	Ch ^a^	K	Kaz_G1	O_G1	O_G2	T_G1	T_G2
Ch	0	**0.018** ^b^	**0.033**	0.236	**0.023**	**0.041**	0.055
K	no	0	**0.038**	0.240	**0.024**	**0.047**	0.052
Kaz_G1	no	no	0	0.252	**0.041**	**0.034**	0.078
O_G1	yes	yes	yes	0	0.232	0.228	0.275
O_G2	no	no	no	yes	0	**0.034**	0.047
T_G1	no	no	no	yes	no	0	0.077
T_G2	yes	yes	yes	yes	yes	yes	0

^a^ Region/group abbreviation: Ch—Chelyabinsk, K—Kurgan, T—Tyumen, O—Omsk, Kaz—North Kazakhstan, T_G1—group 1 in Tyumen, etc.; ^b^ based on ${dif}_{KW}$, distances with no statistically significant extent of differentiation between the corresponding collections are shown in bold; rejection (acceptance) of the hypothesis of “no differentiation” took place at the *p* < 0.01 (*p* > 0.05) level.

## Data Availability

The data used in this study are available from the corresponding author upon reasonable request.

## Supplementary Materials

The following supporting information can be downloaded at https://www.mdpi.com/article/10.3390/jof12100774/s1, Figure S1: UPGMA dendrogram of relationships among virulence phenotypes of *P. triticina* identified in the Tyumen region in 2025.
