# Supplementary material for: Virulence Variability of Puccinia triticina in the Western Region of the High-Latitude Asian Spring Wheat Belt
Source: J Fungi (Basel). 2026 Oct 15;12(10):774. doi: 10.3390/jof12100774 (PMC13706111; doi:10.3390/jof12100774)
Supplement: Supplementary file 1 [file jof-12-00774-s001.zip › jof-4531354-supplementary.pdf]

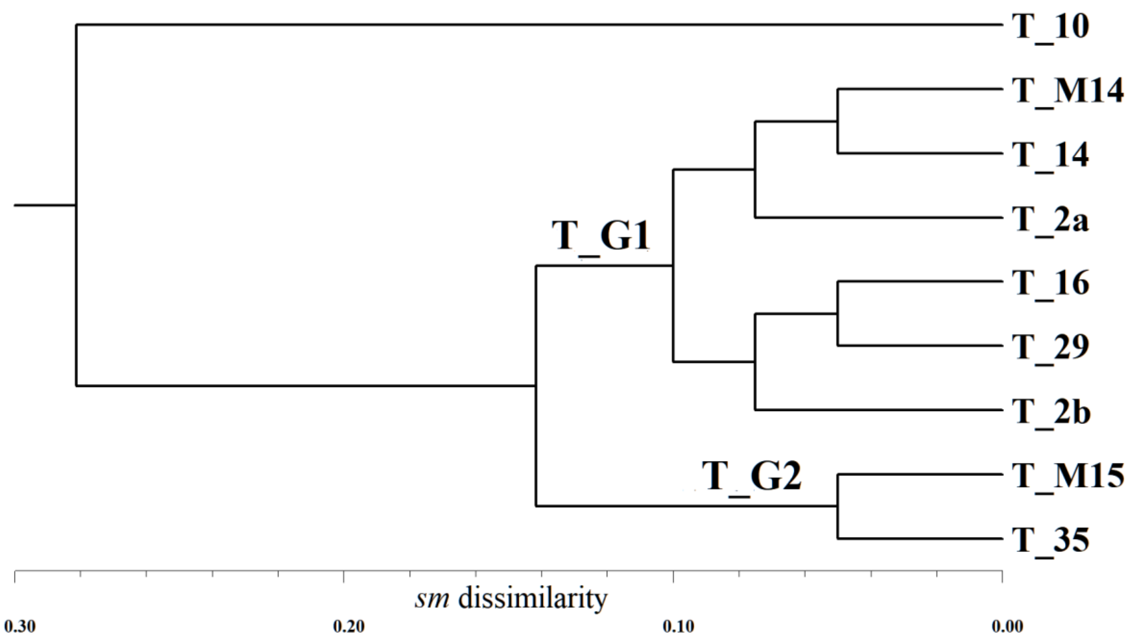

Figure S1. UPGMA dendrogram of relationships among virulence phenotypes of *P. triticina* identified in the Tyumen region in 2025. The phenotype designations include the region (T – Tyumen) and accession number; for example, T_10 means phenotype from Tyumen collected in wheat accession No 10 according to Table 1, T_M14 means phenotype from Tyumen collected in wheat accessions that belong to group M14. The group composition of wheat accessions corresponds to the descriptions in Fig. 2: **T_M14** - No 3-8, 11-13, 15, 17, 24, 26, 27, 30, 32, 36; **T_M15** - No 22, 28, 37.
